# Patients' Preference for Physician Attire in the Internal Medicine Outpatient Department

**DOI:** 10.1155/2023/2992888

**Published:** 2023-01-09

**Authors:** Yiyan Zou, Yiyou Wang, Yang Song, Sen Liu, Zhiyuan Zhang, Limei Wang, Jieshi Zhang, Ruixuan Geng, Zhibo Zheng, Yeye Chen

**Affiliations:** ^1^Department of International Medical Services, Peking Union Medical College Hospital, Chinese Academy of Medical Sciences, Beijing, China; ^2^Department of Thoracic Surgery, Peking Union Medical College Hospital, Chinese Academy of Medical Sciences, Beijing, China

## Abstract

**Objective:**

To investigate patients' preference and the attitude towards physician attire in an internal medicine clinic in China.

**Methods:**

This study was conducted from 1 January 2021 to 30 June 2021 in a tertiary care hospital in China. We surveyed 126 patients in the hospital with 6 sets of pictures of commonly worn physician attires in the hospital setting with a two-part questionnaire. The first part listed respondent demographics to collect basic information. The second part of the questionnaire was administered to adult patients who received care in the internal medicine clinics (outpatients). Survey forms collected demographic data (age, gender, patient age, education, marital status, and employment status), asked questions regarding 6 specific attires (scrubs, scrubs and white coat, casual, casual and white coat, business suit, and formal and white coat), and behavioral items (professional, responsible, reliable, knowledgeable, succession rate, and medical safety), finally, to assess the preference of attire on overall perception.

**Results:**

Scrubs and white coat scored the highest through 6 domains about physicians' attire (professional, reliable, responsible, knowledgeable, medical safety, and succession rate, *p* < 0.05 for all comparisons). A casual suit without a white coat was the least preferred across the surveyed attributes. There was a significant preference gap between wearing a white coat and not wearing a white coat (*p* < 0.001). Physician attire to white coat was considered as more professional, reliable, responsible, knowledgeable, having greater medical safety, and a higher success rate than attires without white coat.

**Conclusion:**

Patients felt that the physician wearing a white coat was better than other attires. Scrubs and white coat was the most popular attire. Hospital or related authorities may promote the standardized wearing of white coats, leading in a greater patient-physician relationship.

## 1. Introduction

Patient-physician relationship is the key to high-quality healthcare [1]. Although many factors may affect the relationship, the most easily modifiable variable is physicians' attire [2]. It is the requirement of effectiveness in patient-physician communication, which also reflects changes from disease centered to patient-centered medical care service.

Multiple studies have shown that patients' perceptions about physician attire are influenced by several factors like physician's sex, specialty, medical practice setting, patient's geographic area, age, specific disease, etc. [2–4]. For example, patients in the intensive care unit (ICU) may prefer scrubs and white coat with a name tag [5]. Patients from the psychiatric clinic may think that physicians with casual clothing are more friendly, while physicians with white coat are not easy to get along [6]. Data from several studies have reflected that patients' perceptions may impact on patient-physician relationship, including trust, satisfaction, evaluation, and feedback. Few studies have been conducted on patient preferences or attitude to physician attire in China. Due to Chinese culture, it may differ from the previous result which is found in other country. Thus, we carried out the survey beginning with internal medicine patients in a hospital. The article aimed at investigating patients' preference for various forms of physician attire and the attitude towards physician attire in the internal medicine outpatient setting.

## 2. Methods

### 2.1. Setting and Sample

Members of the research team visited an internal medicine outpatient department and asked nursing staff to recommend eligible adult patients. Eligible patients were those who were residents of China, were 19 to 100 years of age, spoke and understood Chinese, and possessed sufficient visual, hearing and cognitive abilities to read, to listen to instructions, and to respond to questions. Interested patients notified our principal investigator directly or spoke to one of the staff nurses about participating in the study. After having the patient review the information letter and allowing opportunity for questions, two members of the research team obtained written informed consent and demographic data.

### 2.2. Data Collection Procedures

**Figure 1:**
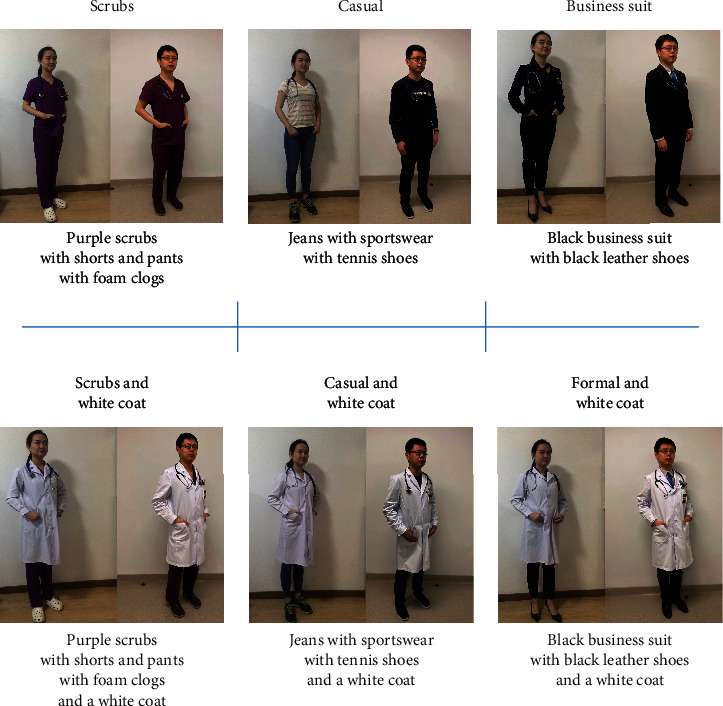
Physician's attire.

Data were collected between 1 January 2021 to 30 June 2021 from 126 adult patients who consented to participate. Following collection of demographic data (i.e., age and gender), participants were presented with photographs of the same physician wearing six different attire styles, colors, and matches (Figure 1).

For each participant, photographs were asked to scan a code to finish the questionnaire online within 15 minutes. If the patients are in need during the process to complete the questionnaire, nursing staff will help at any time.

This study was approved by the Institutional Review Board at the hospital. The need for informed consent by respondents was waived by the Institutional Review Board because of voluntary participation and anonymity of the data collected.

### 2.3. Instrumentation

The valid and reliable MNIS (Cronbach's*α*: 0.991; KMO: 0.932), codeveloped by Albert et al. [7], was used by participants to evaluate physician appearance in each uniform according to 6 traits associated with physician professional image. The questionnaire consisted of 12 questions and included photographs of a male and a female physician in various forms of attire. Survey forms collected demographic data (age, gender, patient age, education, marital status, and employment status), asked questions regarding 6 specific attires (scrubs, scrubs and white coat, casual, casual and white coat, business suit, and formal and white coat), and behavioral items (professional, responsible, reliable, knowledgeable, succession rate, and medical safety), finally, to assess the preference of attire on overall perception. All responses were scored on a five-point Likert's scale with 1 = Strongly Disagree, 3 = Neutral, and 5 = Strongly Agree. Respondents took a survey form and returned it to the staff after completion. The process of writing was not monitored. The survey had no personal identifying information. The collected survey forms were aggregated, and responses were analyzed.

#### 2.3.1. Statistical Analyses

SPSS version 20.0 and GraphPad Prism 7 was used for statistical analyses. The ranking of the six different attires was presented as means and standard deviations (SD). White coat preferences were analyzed by the mean values between the white coat group and the nonwhite coat group using paired *t*-test. Variance analysis is used to know the specific preference among the white coat groups.

## 3. Results

### 3.1. Subject Characteristics

From 1 January 2021 to 30 June 2021, a total of 164 surveys were collected with patients from a tertiary care hospital in China, of which 126 surveys were eligible for analysis. 61.1% of them are female. The age of the 126 patients ranged from 19 to 83 years. Most of the patients are married. Of these, 61.1% were female, and 61.2% had bachelor degrees or above (Table 1).

### 3.2. Preference of Physician Attire

**Figure 2:**
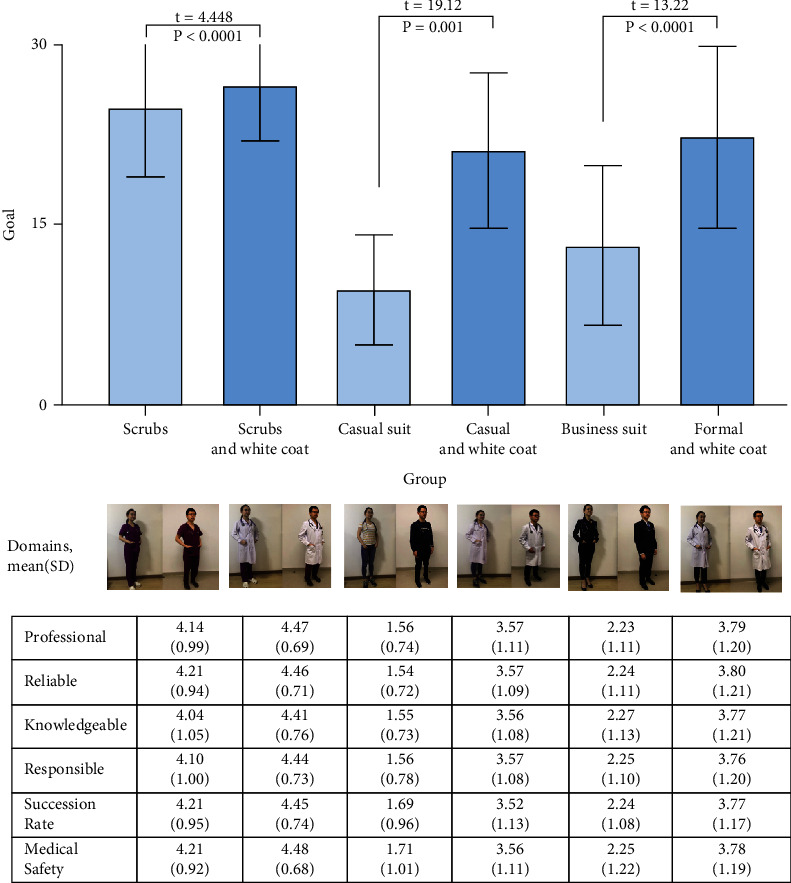
Preferences of attire types, overall and by perceived physician qualities. The columns show the overall average composite rating score for the six female and male attire types (pictures) from left to right: scrubs, scrubs and white coat, casual suit, casual and white coat, business suit, business suit and white coat. The individual results of how per patient feels about physician quality (6 domains) and attire type appear in the following table.

Physicians' attire is shown in Figure 1: scrubs, scrubs and white coat, casual, casual and white coat, business suit, and formal and white coat. In the research, respondents rated “scrubs and white coat” as the most preferred attire, while casual dressing ranked last. When comparing the different attires between white coat and nonwhite coat, patients preferred the white coat group clearly (*P* = 0.01, Figure 2).

### 3.3. Comparison between White Coat and Nonwhite Coat Groups

**Figure 3:**
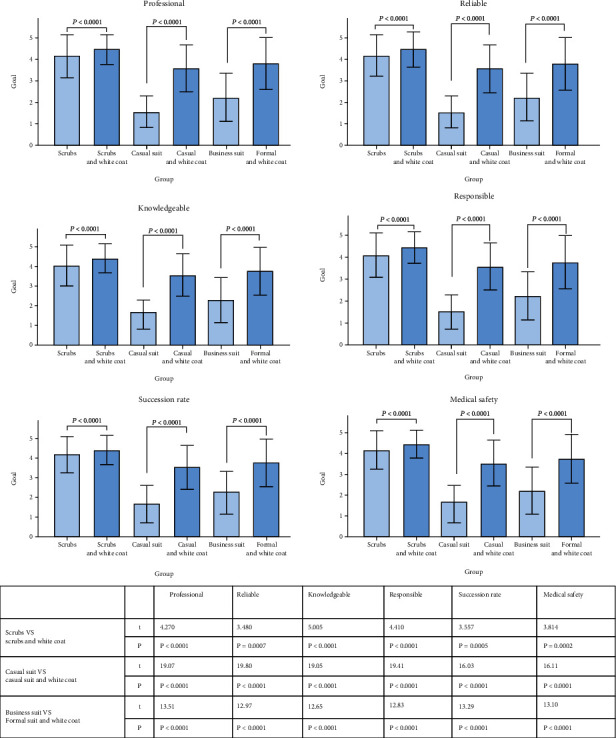
Physician attire preferences across domains.

Figure 3 Across 6 domains (professional, reliable, knowledgeable, responsible, success rate, and medical safety) on how patients feel about the physician, scrubs and white coat had the highest score. Overall, the groups with white coat scored higher than the groups without white coat (*P* < 0.01).

### 3.4. Comparison among the White Coat Groups

**Figure 4:**
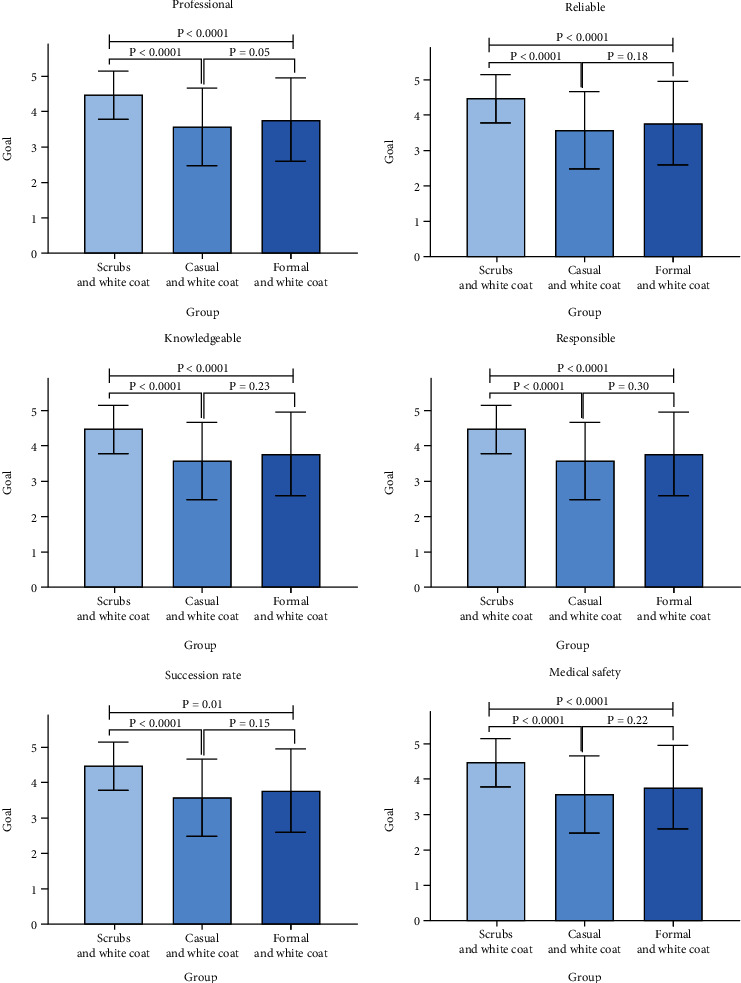
Comparison among white coat groups.

Figure 4 compares three groups (scrubs and white coat, casual suit and white coat, and formal and white coat) among the 6 domains. The result shows no difference between casual and white coat and formal and white coat (*P* > 0.05).

## 4. Discussion

In this study, 126 outpatients in a tertiary care hospital in China provided data on patients' preference about physician attire. The order of preferences is scrubs and white coat, scrubs, formal and white coat, casual and white coat, business suit, casual suit. Respondents preferred scrubs and white coat in every domain, which is consistent with a related study [7]. Among 6 attires, scrubs and white coat was the most preferred in which they may think that the white coat looks more professional, reliable, responsible, and knowledgeable and can strengthen their confidence about the physician, even his or her clinical ability, so that they may trust the physician's medical safety and success rate, and the result is similar to the finding reported by Jennings et al. [4]. In our thought, white coat is usually considered as the professional dress while the casual suit is the least popular suit. There is a possibility which many people think that scrubs is a symbol of good practiced and professionalism, which may come from the spread of television shows. Scrubs is often connected with an emergency, so that patients may think that physicians who wear scrubs are able to deal with clinical problems. It helped the audience build their perception about the physician image. Thus, it is not hard to explain that scrubs and white coat had the highest score. On the contrary, casual suit got the least scores, and the finding varies a lot in different clinic settings. Children or psychic patients prefer casual attires most because it reflects a more compassionate rather than authoritative image [8]. While our study is carried in the outpatient department, the first impression is important to build the physician-patient relationship. Regardless of the physician's appearance, attributes such as demeanor, empathy, tone of voice, hygiene, and even smiling will shape a patient's perception of his or her doctor [9–11]. Patients will not recognize the casual attire due to“white coat”or “scrubs” traditional image of physician.

Comparing with white coat groups and nonwhite groups, white coats were preferred in every domain (*P* < 0.0001). Respondents' predilections for the white coat are consistent with published results, which denote the coat as symbolic for a clean, competent, and professional physician [12].

However, when casual attire or business attire was worn under a white coat, the patient's perception of physician images declined, suggesting that the clothing a physician wears under their white coat is important as well. De Lott et al. [13] showed that formal dress and white coat are overall preferred, while casual attire has the least score among all attires in the ophthalmology department. There are similarities as well as differences [14]. Not surprisingly, some preferences varied by age, practice type, and clinical department [4, 7]. Taken together, these findings point to patient expectations for a physician “uniform”, and patients have an overall preference for scrubs and white coat.

Although there is a clear patient preference for white coat, some physicians eschew white coat out of concern that it may induce anxiety or fear or may pose an infection risk [13]. Different from this perception, in this study, white coat may be considered kinder than nonwhite coat as patients think that physicians wearing white coat are reliable and kind. As we all know, not only physicians would wear white coat but also in many working scenes, so that it may increase the public concern about the risk of infection about the white coat despite the lack of no evidence at present [15].

This is the first study in China, which can provide some data for future study. Secondly, we used photographs of models taken in a standardized manner (e.g., identical postures and facial expressions) rather than relying on descriptions of attire to assess preferences. Nevertheless, in this study, respondents have indicated a strong preference for physicians in scrubs and a white coat and have shown a strong intention to trust, to comply with their succession rate, and to medical safety (*P* < 0.0001). One of the proven and changeable factors that contribute to the first impression and overall patient trust and confidence is the attire of the physician. In the urban internal outpatient setting, we observed modest preferences for the scrubs and white coat, and attire does influence how our patients perceive their physicians' character and abilities; therefore, future endeavors might examine broader influences on the patient-physician relationship such as age and different clinic setting.

This study has its limitations. Firstly, we could only solicit preferences from patients that agreed to take our survey. Those with more significant vision loss may have been less likely to participate, which may bias our results. Secondly, the sample amount is not large enough and may not reflect patients' preference condition about physician attire. Thirdly, we did not solicit open-ended responses for attire preferences, potentially limiting explanations or responses.

## 5. Conclusion

Physician attire is an important and modifiable factor that influences patient satisfaction with medical care. Patients and visitors to an internal medicine clinic in this study were in favor of doctors wearing white coat, especially scrubs and white coat. We recommend that general physician wears a white coat during patient care encounters. We can carry out future studies to learn about their preferences in different clinical settings and to explore the influencing factors. This is particularly important if this attire results in better adherence and thus positive health outcomes. Further study to assess the general liability of these findings is in need.

## Figures and Tables

**Table 1:** Respondent demographics.

Characteristics	Number of patients (*N* = 126)	Percentage (%)
Gender		
Male	49	38.9
Female	77	61.1
Age, years		
≤35	36	28.6
36-45	31	24.6
46-60	36	28.6
61-75	21	16.7
>75	2	1.59
(mean ± SD)	45.32 ± 14.339	
Marital status		
Single	19	15.1
Married	105	83.3
Widow	1	0.8
Divorced	1	0.8
Education		
Primary or lower	6	4.8
Junior high school	14	11.1
Senior high school	29	23.0
Bachelor's degree	54	42.9
Graduate degree	23	18.3
Employment status		
Studying	5	4.0
Employed	66	52.4
Freelance	26	20.6
Unemployed	7	5.6
Retired	22	17.5

## Data Availability

The data that support the findings of this study are available from the corresponding author upon reasonable request.
